# Adrenal Incidentaloma: Challenges in Diagnosing Adrenal Myelolipoma

**DOI:** 10.1177/2324709619870311

**Published:** 2019-08-21

**Authors:** Sreedhar Adapa, Srikanth Naramala, Vijay Gayam, Frank Gavini, Hemant Dhingra, Florette Kimberly Gray Hazard, Narothama Reddy Aeddula, Venu Madhav Konala

**Affiliations:** 1 Kaweah Delta Medical Center, Visalia, CA, USA; 2 Adventist Medical Center, Hanford, CA, USA; 3 Interfaith Medical Center, New York, NY, USA; 4 Saint Agnes Medical Center, Fresno, CA, USA; 5 Stanford University, Stanford, CA, USA; 6 Deaconess Health System, Evansville, IN, USA; 7 Ashland-Bellefonte Cancer Center, Ashland, KY, USA

**Keywords:** adrenal myelolipomas, adrenal incidentaloma

## Abstract

Adrenal myelolipomas (AMLs) are rare benign adrenal tumors, containing adipose and hematopoietic tissue, a result of reticuloendothelial cell metaplasia. Incidence on autopsy has been reported from 0.08% to 0.4%. AMLs are generally considered nonsecretory. The functional aspect of adrenal incidentaloma should be evaluated. In this article, we report a case of a 40-year-old male, who presented with uncontrolled hypertension and renal failure, with imaging revealing an adrenal incidentaloma. He was started on dialysis for acute fluid overload, and workup for pheochromocytoma revealed an elevated serum norepinephrine level of 1181 pg/mL. Free metanephrine and normetanephrine levels were low when checked pre- and post-dialysis. Complete resection of the encapsulated right adrenal mass was performed. Pathology of the adrenal tumor demonstrates an 11.5 × 9.5 × 7.5 cm well-circumscribed, partially encapsulated proliferation of mature adipose tissue with admixed hemopoietic elements consistent with myelolipoma weighing 29.3 g. This case highlights the inclusion of a full metabolic workup for all adrenal incidentalomas, including AML.

## Introduction

Adrenal myelolipomas (AMLs) are rare benign adrenal tumors, containing adipose and hematopoietic tissue, a result of reticuloendothelial cell metaplasia. It is sporadic with an overall incidence reported 0.08% to 0.4% at autopsy previously, compared with increased incidence of 10% to 15% recently due to the widespread use of imaging.^1^ It involves both the genders equally, mostly unilateral, and usually occurs in the fifth and seventh decades of life.^1^ Adrenal incidentalomas (AIs) need mandatory metabolic workup as recommended by guidelines of most endocrine societies. The functional aspect of AI should be evaluated.

## Case Report

A 40-year-old male was evaluated with the chief complaint of a 1-week history of testicular and bilateral lower extremity swelling. He was found to be in a hypertensive emergency with a blood pressure of 234/119 mm Hg and needed intravenous antihypertensive medications to control the blood pressure. Other vital signs on presentation were a temperature of 98.4°F, pulse rate of 100 beats per minute, and oxygen saturation of 92% on room air. Physical examination was significant for decreased air entry on bilateral lung bases, bilateral lower extremity pitting edema, and scrotal edema. Past medical history was significant for hypertension, morbid obesity, chronic kidney disease stage 4, and right adrenal mass, which was diagnosed 5 years prior to the presentation (interval increase in the growth on serial computed tomography [CT] scans). The patient has been prescribed multiple antihypertensives but was noncompliant.

**Figure 1.**
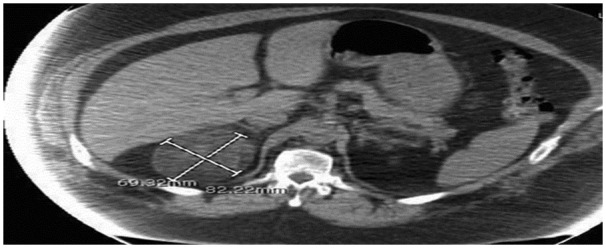
Computed tomography scan of the abdomen showing adrenal myelolipoma measuring 8.2 × 6.9 cm.

Laboratory data showed hemoglobin 8.7 mg/dL, blood urea nitrogen 70 mg/dL, and creatinine 8.7 mg/dL, with glomerular filtration rate 7 mL/min. A CT abdomen/pelvis without contrast revealed an interval increase in the size of hypoattenuating right adrenal mass measuring 9.7 × 7.7 × 6.1 cm compared with 8.2 × 6.9 × 6.4 cm 6 months prior (Figure 1). Other investigations showed increased serum norepinephrine level 1181 pg/mL (80-520 pg/mL), which was 931 pg/mL 6 months prior; also increased serum dopamine level 31 pg/mL (0-20 pg/mL), which was <20 pg/mL 6 months prior. Plasma aldosterone renin ratio and serum cortisol levels were within normal limits (plasma renin activity 0.9 ng/mL/h [0.2-1.6 ng/mL/h], plasma aldosterone level 11.3 ng/dL [4-31 ng/dL], and serum cortisol 12.8 µg/dL [6.7-22.6 µg/dL]). Prior 24 hours urinary metanephrine/normetanephrine levels as well as catecholamine levels were inconclusive (normetanephrine 828 µg/d [110-1050 µg/d], metanephrine 412 µg/d, epinephrine level 12 µg/d [1-7 µg/d], dopamine level 77 µg/d [77-324 µg/d], and norepinephrine 23 µg/d [16-71 µg/d]). The patient was started on hemodialysis in the setting of progressively declining renal function and fluid overload state. Blood pressure was controlled with multiple antihypertensive medications including amlodipine 10 mg daily, hydralazine 100 mg 3 times a day, isosorbide mononitrate 30 mg sustained release daily, minoxidil 5 mg daily, spironolactone 25 mg daily, phenoxybenzamine 10 mg twice a day, and by volume removal on dialysis.

**Figure 2.**
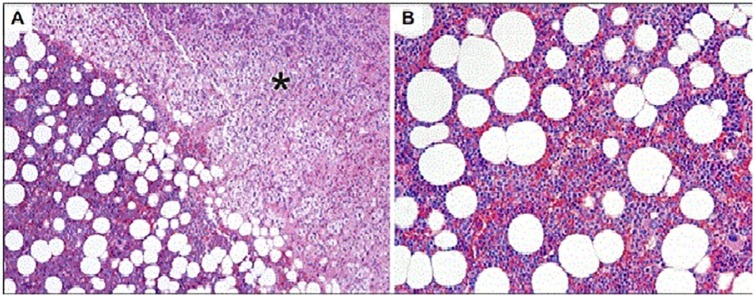
(A) Normal adrenal gland cortex (*) and myelolipoma characterized by hematopoietic elements and adipose tissue (hematoxylin and eosin [H&E], 100×). (B) Myelolipoma hematopoietic elements and adipose tissue reminiscent of normal bone marrow (H&E, 200×).

The patient was transferred to the tertiary care center due to the unavailability of the endocrine surgeon. At the tertiary care center, CT of abdomen and pelvis with contrast revealed heterogeneously enhancing round adrenal lesion on the right measuring 9.9 × 8.7 cm. Average attenuation is approximately 0 Hounsfield units (HU), and lesion enhances to 16 HU on venous and delayed-phase imaging, which is less consistent with pheochromocytoma. There was also little difference in the pre- and post-dialysis levels of metanephrines, which questioned the diagnosis of pheochromocytoma. Given the history of progressively growing adrenal mass, the decision was made to proceed with adrenalectomy. The patient underwent open right adrenalectomy and tolerated the procedure well with no intraoperative hemodynamic instability. A completely encapsulated adrenal tumor was removed intact. Pathology of the adrenal tumor demonstrated an 11.5 × 9.5 × 7.5 cm well-circumscribed, partially encapsulated proliferation of mature adipose tissue with admixed hemopoietic elements consistent with myelolipoma weighing 29.3 g (Figure 2). The patient continues to be dialysis dependent.

## Discussion

Adrenal myelolipoma is a benign tumor originating from the adrenal cortex and is usually nonfunctional. Gierke initially described myelolipoma in 1905, and Oberling coined the term “myelolipomatoses” in 1929.^2^ The tumor is composed of mature adipose tissue admixture with hematopoietic elements. AML involving extra-adrenal locations like thorax, liver, spleen, stomach, mesentery, pelvis, and retroperitoneum have been reported.^3^ Rarely they can be functional or coexist with other endocrine disorders like Cushing syndrome, congenital adrenal hyperplasia (CAH), primary aldosteronism, and pheochromocytoma. CAH has been increasingly reported with AML.^4^ Chronic ACTH over stimulation of adrenals may have a role in patients with bilateral AML and also in patients with untreated CAH with AML.^5^

The comorbidities like hypertension, obesity, diabetes, atherosclerosis, and malignancy have been associated with AML.^6^ AML has been reported with thalassemia rarely and tend to be giant and bilateral, due to increased production of erythropoietin.^7^

The origin of AML is unclear; few postulated mechanisms are extramedullary hematopoiesis, hamartosis, embolism of bone marrow element, metaplasia of reticuloendothelial cells of blood capillaries (infections, chronic stress, inflammation, and necrosis) in adrenal glands, and degeneration of adrenal cortical cells.^7,8^ AML is a clonal tumor based nonrandom X-chromosome inactivation identified on recent cytogenetic studies.^7^

Adrenal myelolipoma is usually asymptomatic and generally has a diameter <5 cm, but can vary from <1 cm to >30 cm. Sometimes the diameter of AML is >10 cm, often described as giant AML. Akamatsu et al described the largest AML without the endocrine disorder, measuring 31 × 24.5 × 11.5 cm and weighing 6000 g.^9^ Boudreaux et al described the largest AML in a patient with CAH, size 34 × 24 × 10.5 cm, weight 5900 g.^10^

The serum catecholamine levels are not reliable for the diagnosis of pheochromocytoma.^11^ The sensitivity and specificity of plasma catecholamines are very low at 78.6% and 70.7%, respectively.^12^ The plasma-free metanephrines or urinary fractionated metanephrines are more sensitive and specific in pheochromocytoma.^13^ The CT scan also showed low Hounsfield units, which are less likely, consistent with pheochromocytoma.^14^

Recent American Association of Clinical Endocrinologists (AACE) disease state clinical review suggested that incidentally discovered adrenal masses require dynamic and static hormonal measurements.^15^ The previous AACE guidelines published in 2009 did not recommend metabolic workup for AML.^16^ Endocrine dysfunction of AML is underappreciated as the review of the literature reveals that 7% in AML and 11% in AI were functional.^8^ AML should not always be excluded from metabolic workup. Endocrine workup is beneficial in AML patient with hypertension, younger patients, diabetes or prediabetes, and those with bilateral AML.^8^ Function AML resection resulted in resolution of hypertension and sequelae in several reports, underestimating the incidence of hormonal abnormality.

Adrenal myelolipoma can be diagnosed in 90% of the cases by ultrasonography, CT, and magnetic resonance imaging.^4^ CT is more sensitive for detection than other imaging modalities.^7^ Ultrasonography appearance can be hyperechoic or hypoechoic depending on the predominance of fat or myeloid cells. Similarly, CT can have a high attenuation with myeloid tissue and low attenuation with a fatty tumor. Magnetic resonance imaging appearance of the tumor demonstrates the high signal intensity and reduced signal intensity depending on the T1-weighted or T2-weighted sequences, respectively. Retroperitoneal fat–containing tumors like teratoma, lipoma, myolipoma, angiomyolipoma, and liposarcoma may mimic AML radiologically.^6,8^

Adrenal biopsy should be considered in the following circumstances as per endocrine literature: (1) hormonal inactivity of the adrenal mass, especially ruling out pheochromocytoma; (2) benign characteristics of an adrenal mass not established on imaging; and (3) biopsy results of adrenal mass would alter the management.^17^

Pathological diagnosis of AML requires the presence of hematopoietic elements and mature adipocytes. Management of AML is dependent on the size of the tumor. Tumors <5 cm are generally asymptomatic and can be monitored intermittently with imaging (1-2 years).^4^ Surgical removal is indicated if the patient is symptomatic, tumor >5 cm, increased risk of rupture, or if malignancy is suspected. Spontaneous retroperitoneal hemorrhage is a well-recognized complication of AML, which is rare.^4^

## Conclusion

This case highlights the inclusion of a full metabolic workup for all adrenal incidentalomas, including AML. The serum catecholamine levels are not reliable for diagnosis of pheochromocytoma, but measured plasma metanephrine levels or urinary fractionated metanephrines are more sensitive and specific in pheochromocytoma.
